# Validity of 2D lateral cephalometry in orthodontics: a systematic review

**DOI:** 10.1186/2196-1042-14-31

**Published:** 2013-09-20

**Authors:** Ana R Durão, Pisha Pittayapat, Maria Ivete B Rockenbach, Raphael Olszewski, Suk Ng, Afonso P Ferreira, Reinhilde Jacobs

**Affiliations:** 1 Department of Dental Radiology, Faculty of Dental Medicine, University of Porto, Porto, Portugal; 2 Oral Imaging Center, OMFS-IMPATH research group, Dept Imaging & Pathology, Faculty of Medicine, University of Leuven, Leuven, Belgium; 3 Department of Surgery, Dentistry School, Pontifical Catholic University of Rio Grande do Sul, Porto Alegre, Rio Grande do Sul, Brazil; 4 Department of Oral and Maxillofacial Surgery, Université Catholique de Louvain, Brussels, Belgium; 5 Department of Dental Radiology, Guy's, King's and St. Thomas' Dental Institute, King's College London, London, UK; 6 Department of Orthodontics, Faculty of Dental Medicine, University of Porto, Porto, Portugal

**Keywords:** Cephalometry, Orthodontics, Systematic review, Reliability, Validity

## Abstract

Lateral cephalometric radiography is commonly used as a standard tool in orthodontic assessment and treatment planning. The aim of this study was to evaluate the available scientific literature and existing evidence for the validation of using lateral cephalometric imaging for orthodontic treatment planning. The secondary objective was to determine the accuracy and reliability of this technique. We did not attempt to evaluate the value of this radiographic technique for other purposes. A literature search was performed using specific keywords on electronic databases: Ovid MEDLINE, Scopus and Web of Science. Two reviewers selected relevant articles, corresponding to predetermined inclusion criteria. The electronic search was followed by a hand search of the reference lists of relevant papers. Two reviewers assessed the level of evidence of relevant publications as high, moderate or low. Based on this, the evidence grade for diagnostic efficacy was rated as strong, moderately strong, limited or insufficient. The initial search revealed 784 articles listed in MEDLINE (Ovid), 1,034 in Scopus and 264 articles in the Web of Science. Only 17 articles met the inclusion criteria and were selected for qualitative synthesis. Results showed seven studies on the role of cephalometry in orthodontic treatment planning, eight concerning cephalometric measurements and landmark identification and two on cephalometric analysis. It is surprising that, notwithstanding the 968 articles published in peer-reviewed journals, scientific evidence on the usefulness of this radiographic technique in orthodontics is still lacking, with contradictory results. More rigorous research on a larger study population should be performed to achieve full evidence on this topic.

## Review

### Introduction

Since the introduction of lateral cephalometric radiography in 1931 by Broadbent in the USA and by Hofrath in Germany, this radiograph and its related analyses have become a standard tool in orthodontic assessment and treatment planning [1-3]. Lateral cephalometric radiographs are systematically collected prior to orthodontic treatment in many European countries [3,4]. Nevertheless, the real value of this imaging technique for diagnosis and planning of orthodontic treatment remains uncertain [2-7]. Some authors showed that an adequate orthodontic diagnosis and treatment plan could not be performed without comparing a lateral cephalometric radiograph before and after orthodontic treatment and that treating skeletal malocclusions without a cephalometric radiograph introduced serious errors [8]. While only a small percentage of the orthodontic treatment planning are modified based on lateral cephalometric radiographic analysis [6], it could adjust some aspects of treatment planning, such as tooth extraction, extract pattern and anchorage features [2,9].

The controversy about the correct use of the lateral cephalometric radiograph is also present in orthodontic textbooks where guidelines for orthodontic imaging are not expressed properly [10]. Several radiographic techniques, like panoramic and full-mouth periapical radiographs, used in orthodontics are found unproductive, since it provides duplicate information [4]. The latter is an important finding as the use of ionising radiation should always be justified and kept ‘as low as reasonably achievable’ and definitely in children as radiographs are often performed at different time intervals during orthodontic treatment [11,12]. Even when there are means to optimise radiation dose of cephalometric radiographs, the primary issue is to justify the decision to take a lateral cephalogram prior to orthodontic treatment [10,11,13-15].

The present systematic review was initiated by the fact that three-dimensional (3D) cephalometric analysis is emerging, while there is still lack of scientific evidence on the validity and reliability of two-dimensional (2D) cephalometric imaging for orthodontic treatment planning [2,3].

Therefore, the aims of this study were to systematically review the available scientific literature and to evaluate the existing evidence about the validation of lateral cephalometric radiograph in orthodontics. This review also studied the accuracy and reliability of lateral cephalograms and its cephalometric analysis.

### Materials and methods

#### Information sources

A comprehensive electronic database search to identify relevant publications was conducted, and the reference lists in relevant articles were searched manually for additional literature. We set no language limitations, although we did not attempt to explore the informally published literature: conference proceedings and abstracts of research presented at conferences and dissertations. The following databases were searched: Ovid Medline (1946 to 11 January 2012), Scopus (to 11 January 2012) and Web of Science (1899 to 11 January 2012).

#### Search strategy

We developed the search strategy with the help of an information specialist at King's College London Dental Institute in London. The searches did not have a date limit and were not restricted to particular types of study design. The search strategy focused on the following terms: Cephalometr* and (orthodontic* or ‘orthodontic treatment planning’) and (‘efficacy’ or ‘reproducibility’ or ‘repeatability’ or ‘reliability’ or ‘accuracy’ or ‘validity’ or ‘validation’ or ‘precision’ or ‘variability’ or ‘efficiency’ or ‘comparison’) not (‘Cone-Beam Computed Tomography’ or ‘Three-Dimensional imaging’ or ‘Cone Beam Computed Tomography’ or ‘Cone Beam CT’ or ‘Volumetric Computed Tomography’ or ‘Volume Computed Tomography’ or ‘Volume CT’ or ‘Volumetric CT’ or ‘Cone beam CT’ or ‘CBCT’ or ‘digital volume tomography’ or ‘DVT’ or ‘Spiral Computed Tomography’ or ‘Spiral Computer-Assisted Tomography’ or ‘Spiral Computerized Tomography’ or ‘spiral CT Scan’ or ‘spiral CT Scans’ or ‘Helical CT’ or ‘Helical CTS’ or ‘Helical Computed Tomography’ or ‘Spiral CAT Scan’ or ‘Spiral CAT Scans’ or ‘3D’ or ‘3-D’ or ‘three dimension*’).

#### Study selection

At the first stage, two reviewers (experienced dento-maxillofacial radiologists) independently screened the titles of the retrieved records, and only the titles related to 2D cephalometry, radiographs for orthodontic treatment and tracings were included. Next, the abstracts of the retrieved publications were read by the two observers and categorised according to the study topic. An article had only to be justified by one observer to be included for the second selection phase. All articles of interest in languages other than English were included. Of these two were included, one article was written in Portuguese and another in French. Eligibility of potential articles was determined by applying the following inclusion criteria to the article abstracts: (1) technical efficacy, (2) diagnostic accuracy efficacy, (3) diagnostic thinking efficacy, (4) therapeutic efficacy, (5) patient outcome efficacy or any combination of the previous items as published by Fryback and Thornbury [16]. The other inclusion criteria were (1) accuracy, (2) reliability, (3) validity of lateral cephalometric radiograph, (4) landmark identification on tracings (intra- and inter-observer errors) and (5) the effect of using 2D cephalometry on the orthodontic treatment plan.

Diagnostic accuracy efficacy was defined as follows:

1. Observer performance expressed as overall agreement, kappa index or correlation coefficients

2. Diagnostic accuracy as percentage of correct landmark identification and further tracing analysis, validity and effectiveness of cephalometry in orthodontic treatment planning

3. Sensitivity, specificity or predictive values of landmark identification

Diagnostic thinking efficacy was defined as follows:

1. Percentage of cases in a series in which images were judged ‘helpful’ for the diagnosis

2. Difference in clinicians' subjective estimated diagnosis probabilities before and after evaluation of the cephalogram

Therapeutic efficacy was defined as follows:

1. Percentage of times the image was judged helpful in planning management of the patients in a case series

2. Percentage of times therapy-planned pre-visualization of a lateral cephalogram needed to be changed after the image information was obtained

3. Percentage of times clinicians prospectively stated therapeutic choices needed to be changed after evaluating a cephalogram

4. Whether different analyses lead to different decisions on treatment planning

5. Intra- and inter-observer identification errors

6. Reliability of landmark identification

The analysis had to be based on primary materials or comprise a review on efficacy. When an abstract was considered by at least one author to be relevant, it was read in full text. At the second stage, the full texts were retrieved and critically examined. Reference lists of publications that had been found to be relevant in the first stage were hand-searched, and articles containing the words ‘cephalometry’ , ‘lateral cephalometric radiography’ , together with ‘treatment planning’ , ‘orthodontic radiographs’ , ‘landmark identification’ and ‘error’ were selected. Book chapters and reviews were excluded since the aim of this systematic review was to evaluate primary studies.

#### Data extraction

Data was extracted with the aid of protocol 1 (Table S1 in Additional file 1). It was established by reading the relevant literature on how to critically evaluate studies about diagnostic methods. To minimise bias, two observers independently evaluated the quality and validity of original studies according to the quality assessment of diagnostic accuracy studies tool using protocol 2 (quality assessment of studies of diagnostic accuracy included in systematic reviews - QUADAS) (Table S2 in Additional file 1) [17]. When there was any disagreement concerning the relevance of an article, it was resolved by a discussion between the two reviewers. Each observer presented their arguments, and further discussion was held until a consensus was reached. Before the assessment, the protocols were tested for ten publications. A further five publications were read to calibrate the two reviewers regarding the criteria in protocol 2. Only publications that were found to be relevant to the reviewer in both protocols 1 (diagnostic efficacy) and 2 (level of evidence) were ultimately included. The quality and internal validity (level of evidence) of each publication was judged to be high, moderate or low according to the criteria in the following subsection [18].

#### Levels of evidence and criteria for evidence synthesis

##### High level of evidence

A study was classified with high level of evidence if it fulfilled all of the following criteria:

● There was an independent blind comparison between test and reference methods.

● The population was described so that the status, prevalence and severity of the condition were clear. The spectrum of patients was similar to the spectrum of patients on whom the test method will be applied in clinical practice.

● The results of the test method being evaluated did not influence the decision to perform the reference method(s).

● Test and reference methods were well described concerning technique and implementation.

● The judgments (observations and measurements) were well described considering diagnostic criteria applied and information and instructions to the observers.

● The reproducibility of the test method was described for one observer (intra-observer performance) as well as for several (minimum 3) observers (inter-observer performance).

● The results were presented in terms of relevant data needed for necessary calculations.

##### Moderate level of evidence

A study was assessed to have a moderate level of evidence if any of the above criteria were not met. On the other hand, the study was assessed not to have deficits that are described below for studies with a low level of evidence.

##### Low level of evidence

A study was assessed to have a low level of evidence if it met any of the following criteria:

● The evaluation of the test and reference methods was nonindependent.

● The population was not clearly described, and the spectrum of patients was distorted.

● The results of the test method influenced the decision to perform the reference method.

● The test or the reference method or both were not satisfactorily described.

● The judgments were not well described.

● The reproducibility of the test method was not described or was described for only one observer.

● The results could have a systematic bias.

● The results were not presented in a way that allowed efficacy calculations to be made.

#### Rating conclusions according to evidence grade

The scientific evidence of a conclusion on diagnostic efficacy was judged to be strong, moderately strong, limited or insufficient depending on the quality and internal validity (level of evidence) of the publications assessed [18,19]:

● Strong research-based evidence: at least two of the publications or a systematic review must have a high-level of evidence.

● Moderately strong research-based evidence: one of the publications must have a high level of evidence and two more of the publications must have a moderate level of evidence.

● Limited research-based evidence: at least two of the publications must have a moderate level of evidence.

● Insufficient research-based evidence: scientific evidence is insufficient or lacking according to the criteria defined in the present study.

#### Synthesis of evidence

The results of this review were described narratively. No meta-analyses were attempted because of lack of original studies.

### Results

**Figure 1:**
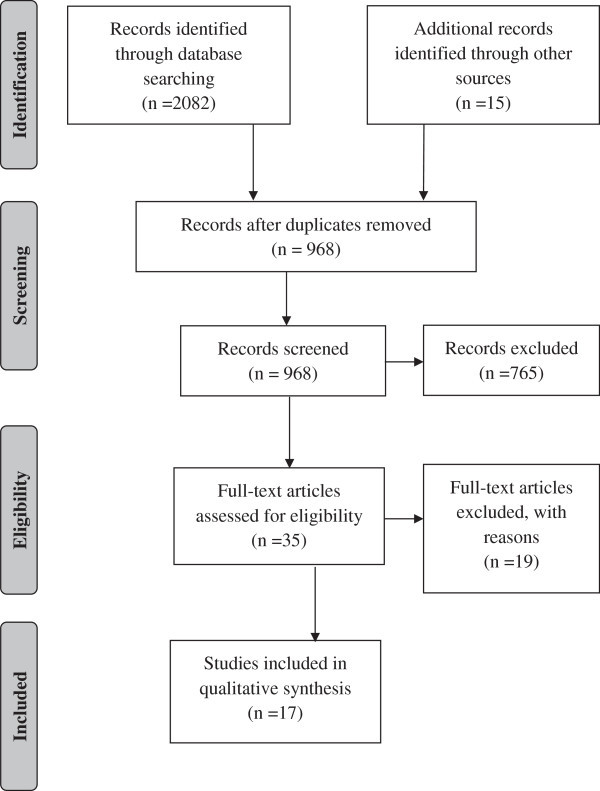
**Methodology followed in the article selection process (adapted from Moher et al.**[20]**).**

The number of articles reviewed in each phase to perform this systematic review is presented in the PRISMA flow diagram (Figure 1) [20]. The initial search revealed 784 articles listed in Medline (Ovid), 1,034 in Scopus and 264 articles in the Web of Science. The second stage of the search protocol was to retrieve the reference lists of the selected articles, which yielded 14 additional articles of interest. After excluding 1,128 duplicates, 968 articles remained for review. In the first phase selection, the observers screened the articles by reading titles and abstracts. Articles that were not eligible because of irrelevant aims and were not directly related to this systematic review were excluded, thus 203 articles remained for further reading. Thirty-five articles were assessed for eligibility.

After screening all the articles using protocols 1 and 2, 17 articles met the inclusion criteria and were selected for qualitative synthesis and appraised to present some level of evidence. All articles that remained after screening passed the qualitative synthesis.

These 17 articles were categorised by topics as follows: 7 studies on the role of cephalometry on the orthodontic treatment planning, 8 studies on cephalometric measurements and landmark identification and 2 studies on cephalometric analysis.

#### Role of cephalometry on the orthodontic treatment planning

Seven articles related to the importance and contribution of cephalometry to orthodontic treatment planning were found (Table 1). Six of the publications were found to have low levels of evidence [2-4,6,9,10] and one classified as moderate level of evidence [7].

**Table 1:** Publications related to the importance and contribution of cephalometry on the orthodontic treatment planning

**Authors (year)**	**Aim of the study**	**Observers**	**Subjects**	**Design of the study**	**Statistical method**	**Results according to authors**	**Level of evidence**
Silling et al. [9]	Assess usefulness of cephalometric analysis	24 orthodontists	6 patients	Stratified random design: 12 orthodontists analysed 6 patients with cephalograms and 12 orthodontists studied 6 patients without cephalogram	Not referred	Class I patient: disagreement on extractions, anchorage and growth potential decisions	Low
						No need for lateral cephalometry, except for atypical class II division 1 patients, by 4 orthodontists	
						Anchorage problems SS between patients with and without lateral cephalogram	
Bruks et al. [6]	Evaluation of lateral cephalometric and panoramic radiography	4 dentists and senior orthodontist	70 patients	Clinical evaluations and treatment plan by 4 dentists:	Descriptive statistics and statistical analyses with computer software. Kruskal-Wallis test to evaluate differences between groups	Impact on diagnosis relating to the ordering sequence of cephalogram: first choice, 68%; second choice, 73%; third choice, 80%	Low
				1. Study casts + photographs		93% of cases: same treatment plan before and after radiographic analysis	
				2. Adding radiographs			
Pae et al. [7]	Examine the link between lateral cephalograms and occlusal trays	16 orthodontists	80 patients	T1: casts evaluated; T2 (1 week later): casts + lateral cephalograms	Rash model, regression plots, two-way ANOVA, *post hoc* multiple comparison Bonferroni and paired *t* test	Class II division 2 patients: 126 extractions planned at T1; 80 at T2	Moderate
						A lateral cephalogram influenced degree of severity, but not the difficulty of treatment	
Nijkamp et al. [3]	Influence of lateral cephalometry on treatment plan	10 post-graduatetrainees and 4 orthodontists	48 patients	Randomised crossover design - T1: casts, T2 (1 month after): with lateral cephalometry and tracing, and T3 and T4 (repeated after 1 and 2 months)	Overall proportion of agreement	Consistency of treatment plan was NS between the use only of dental casts or with additional cephalometry	Low
						Influence of cephalometrics on orthodontic treatment planning: NS	
Devereux et al. [2]	Influence of lateral cephalometry on treatment plan	114 orthodontists	6 patients	3 groups: (a) no lateral cephalogram and tracings, (b) some with lateral cephalogram and tracings and (c) all with lateral cephalogram and tracings	Chi-square and binary logistic regression	Treatment plan changed for extraction pattern (42.9%), anchorage reinforcement (24%) and decision to extract (19.7%)	Low
						Class I patient: lateral cephalogram less times ordered. Only patients where treatment plan changed after its analysis	
						NS impact of cephalometrics on treatment plan	
Atchison et al. [4]	Determine quantitatively the diagnosis and treatment plan information after radiograph evaluation	39 orthodontists	6 patients	A 2-h interview for diagnosis and treatment planning of 6 cases. Study cast, intra- and extra-oral photographs, tracing and clinical findings available.	Analysis of variance with repeated measures and covariance, homogeneity value and descriptive statistics	98% of cases: at least one of the radiographs unproductive	Low
				A radiograph only if judged helpful		3/4 of radiographs did not provide information to change diagnosis and treatment plan	
Atchison et al. [10]	Identify selection criteria for ordering orthodontic radiographs	39 orthodontists	6 patients	A 2-h interview for diagnosis and treatment planning of 6 cases. Study cast, intra- and extra-oral photographs, tracing and clinical findings available	Not referred	14.4% of radiographs ordered for skeletal relationship of the jaws	Low
						Lateral cephalograms accounted for 34% of required information	
						26% of all ordered radiographs produced modifications on diagnosis or treatment plan	
						Pretreatment lateral cephalogram required in all patients needing orthodontic treatment	

NS, non-significant.

#### Cephalometric measurements and landmark identification

Only eight articles were selected as eligible in this category (Table 2). Five publications presented a moderate level of evidence [21-25], while the other three were identified as having a low level of evidence [5,26,27].

**Table 2:** Publications concerning landmark identification

**Authors (year)**	**Aim of the study**	**Observers**	**Subjects**	**Design of the study**	**Statistical method**	**Results according to authors**	**Level of evidence**
Baumrind and Frantz [21]	Quantification of errors in landmark identification	5 observers	20 lateral skull radiographs	Observer identified 16 cephalometric landmarks on a transparent plastic template	Mean, standard deviation and standard errors	Least reliable landmarks: gonion and lower incisor apex	Moderate
	Effects of errors on angular and linear measurements						
Kvam and Krogstad [27]	Evaluation of measurements in lateral cephalograms.	18 observers	3 lateral skull radiographs	Hand cephalometric analysis made by each participant, 8 angles measured	Mean and standard deviation	16 out of 24 angular measurements: less variability in post-graduates than students	Low
	Assess influence of knowledge and impact of angular errors					In 7 measurements, no difference was observed	
						Post-graduates' tracings used for diagnostic purposes	
						Standard deviation of students greater than post-graduates	
Haynes and Chau [22]	Evaluation of landmark identification on Delaire analysis	2 observers	28 lateral skull radiographs	Establish a co-ordinate system for measurement on tracings	Mean deviation	Intra-observer: NS differences between values of T1 and T2 tracings	Moderate
	Comparison with data of conventional cephalometry			Radiographs were traced twice by each observer (3 to 4 weeks)		Inter-observer: differences between the averaged mean values on tracings were NS for either *x* or *y* co-ordinates	
Ahlqvist et al. [26]	Study the magnitude of projection errors on measurements in cephalometry	1 observer	A patient was modelled	Computer software designed to allow movement of model on the 3 axes. The magnitude of errors was studied by a diagram	Measurement errors studied by a diagram with the relative length of distances between modelled landmarks	Less than 1% error on length measurements if head is rotated up to 5°	Low
	Study the effects of incorrect patient position on linear measurements					Head rotated more than 5° the error is increased	
Houston et al. [23]	Evaluate errors at various stages of measurements in cephalometric radiograph	4 observers	24 lateral cephalograms	2 radiographs of the same patient	Analysis of variance	Error variance is small (radiograph and tracing) when compared with the variance among groups	Moderate
				Radiographs traced on acetate sheet by each observer at T1/T2 (1-week interval)		SNA has a higher tracing variance than SNB due to the difficulty to identify point A	
Kamoen et al. [24]	Determine errors involved in landmark identification and its consequence to treatment results	4 observers	50 lateral cephalograms	Items studied: (1) accuracy of digitiser, (2) intra- and inter-observer digitising errors and (3) intra- and inter-observer tracing errors	(1) Levene's test for homogeneity of variances, (2) one-way ANOVA and (3) Levene's test for homogeneity	(1) NS variances of co-ordinates for landmark at different positions on the digitiser. (2) NS intra- and inter-observer differences in digitisation. (3) S differences in landmarks and in the same landmark on different cephalograms and between observers	Moderate
Tng et al. [25]	Evaluate the validity of dental and skeletal landmarks. Effect on angles and distances.	1 observer	2 lateral cephalograms of 30 dry skulls	Steel balls placed in 15 dental and skeletal landmarks	Mean and standard deviation	7 out of 10 skeletal and 5 dental landmarks were NS (*p* < 0.05)	Moderate
				Two radiographs taken with and without the markers and digitised. Measurements compared		4 angles (SNA-SN/MnP, MxP/MnP and LI/MnP) and 3 distances (N-Me, MxP-Me and Lie to APg) were invalid (*p* < 0.05)	
						Major errors in angles with dental landmarks	
Bourriau et al. [5]	Analyse the influence of film-object distance and type of receptor on landmark identification	53 orthodontists	4 lateral cephalograms of the same patient	19 cephalometric landmarks on each film	Mean	NS difference between 2 imaging receptors neither between 2 cephalograms achieved by 2 equipments (*p* > 0.99)	Low
				2 radiographs performed at an equipment with a 4-m arm and 2 in a 1.50-m arm equipment with 2 different imaging receptors (digital and indirect digital)		Results obtained by cephalometric analysis was judged: ‘very important’ for 20.5%, ‘important’ for 70%, ‘less important’ for 8% and ‘accessory’ for 1 participant	

NS, non-significant; S, significant.

#### Cephalometric analysis

Two publications with low-level evidence were found [28,29]. The studies did not use any reference standards, and the number of observers was not stated. The study designs were also not clearly explained (Table 3).

**Table 3:** Publications on cephalometric analysis

**Authors (year)**	**Aim of the study**	**Observers**	**Subjects**	**Design of the study**	**Statistical method**	**Results according to authors**	**Level of evidence**
De Abreu [28]	Assessment criteria of unanimity for different cephalometric analyses	Not referred	129 patients	Diagnosis performed based on Ricketts, Steiner, Cervera and Coutand cephalometric analyses	Not referred	3 out of 61 cases with similar diagnosis. In 23 cases, 4 analyses achieved similar diagnosis. In 13 cases, 3 different diagnoses were obtained. In 8 cases, the diagnosis was different for class II and class III	Low
Abdullah et al. [29]	Examine accuracy and precision of Steiner analysis for changes on ANB angle, the Pg-NB distance and upper and lower incisor positions	Different orthodontists (not reference to the number)	275 patients	Radiographs traced and analysed by orthodontists according to the Steiner analysis	Paired *t* test, mean and standard deviation	The predicted change in L1 (lower incisor) to NB was underestimated by 0.8 mm. Only the prediction for pogonion and NB showed improvement of the precision (30%)	Low
				Radiographs at the end of treatment (T2) were traced by one observer			

### Discussion

The validity, efficacy and contribution of cephalometry in orthodontic treatment planning remain questionable [2]. In 2002, 90% of orthodontists in the USA routinely performed cephalometric radiographs [3]. This systematic review was performed to assess the validity and reliability of 2D lateral cephalometry used for orthodontic treatment planning as well as the errors that can occur on 2D tracing. Despite the abundant amount of articles found on lateral cephalometry (*n* = 968), it is surprising that the present systematic review could only identify very few studies (*n* = 16, 1.6%) on its validity and reliability. This finding underlines the need for the present study and is an important cross point, considering the fact that we are flooding into 3D cephalometric studies nowadays. Apart from our findings, 2D cephalometry has other specific limitations, such as orthognatic surgery, airway and growth assessment and skeletal maturation. In order to be included in this systematic review, publications had to satisfy pre-defined methodological criteria. Two protocols were used regarding the search strategy, one based on diagnostic methods and the second based on the QUADAS tool [17]. The ‘levels of evidence’ for assessing the quality and internal quality of each publication included in this review - how well the study was designed, how reliable its results appeared to be and the extent to which it addressed the questions posed - were modified according to the Oxford Center for Evidence-Based Medicine levels of evidence for diagnostic methods (CBEM) [18]. Only publications assessed to present a high or moderate level of evidence can form the basis for any scientific conclusions. Ten articles were identified as low level of evidence, five had moderate level and only one showed high level of evidence.

All retrieved articles, assessing the importance and contribution of lateral cephalometric radiograph in orthodontic treatment, concluded that there is no significant difference on treatment planning decision with or without the evaluation of the lateral cephalogram. However, it should be considered that the suitable studies in this review were based on small samples rather than large cohorts representing the entire population. In one study, the sample used was restricted (six patients) [2]. Furthermore, the short time lapse between observations in some studies did not allow a full washout effect, which could lead to the repetition of the results [4,7,10]. The latter bias is further strengthened by the fact that recognition factors were often included, e.g. the possibility of identifying patient by photographic visualisation as part of the examination. On the other hand, in one paper, only dental casts were presented to the observers, which might also lead to error since it does not mimic the clinical situation. Sample bias is also suspected based on the fact that selection of subjects is often poorly described or unclear [2,6,9], like the questions made to the observers that were not stated by any questionnaire [6], and in one article, observers were forced to choose yes/no answers, which again do not perfectly simulate the reality [3].

In the two articles by Atchison et al. there was the possibility to identify patients as well as sample size was very restricted (six patients). There was no repetition of the questionnaire to test the variability between answers [4,10]. When it comes to the validity and reliability of cephalometric analysis, several errors should be considered: landmark identification, tracing and measuring, and magnification of certain anatomical structures.

Landmarks placed in anatomically formed edges are easier to identify, while some landmarks placed on curves are more prone to error. The gonion and lower incisor apex are the least consistent landmarks [21]. Furthermore, landmarks such as point A have a higher variance than others like point B because of wider variation and anatomical localisation of point A [23]. Dental landmarks tend to have poorer validity than skeletal landmarks. Also, when landmarks are located on a curve like point A, point B or pogonion, the error is larger [25]. The evidence shows that landmark identification is a great source of error in 2D lateral cephalometry [24]. Major errors in angles with dental landmarks may occur [25]. In addition, different levels of knowledge and experiences between the observers also lead to varying results on landmark identification. In a study using 18 observers, in which 13 were dental students and 5 were post-graduate students, post-graduate's revealed lower intra-observer tracing variance than dental students [27]. Patient positioning during the procedure is also very important to avoid errors on measurements and landmark identification [23,26]. The publication of Ahlqvist et al. [26] was assessed with a low level of evidence because there was only one observer. A similar classification occurred for Bourriau et al. [5], intra-observer agreement could not be evaluated and the number of radiographs (*n* = 4) used was very low. Kvam and Krogstad's [27] publication also used a limited number of subjects (*n* = 3). The choice of the observers also plays an important role on the results. Eighteen observers, in which 13 were dental and 5 were post-graduate students, participated in their study [27]. The latter can also bias results because of the distinct level of education and expertise due to the lack of experience of the observers.

Regarding the influence of magnification, Bourriau et al. [5] could not identify significant differences between equipment with a 4-m distant cephalometric machine and a 1.5-m distant cephalometric arm. Despite that, it should be considered that distance varying between the X-ray source and the image receptor will always cause a degree of magnification, the larger the distance, the lower the magnification. A focus object distance of 4 m in 2D cephalometric equipment is usually favoured for the reduced radiation burden and lack of enlargement, while an equipment with 1.5-m arm has a direct advantage of being compact and integrated in a multimodal system as well as having an increased resolution. On the other hand, panoramic equipment with a cephalometric arm at a 1.5-m distance may present shortcomings in enlargement factors and superimposition of the bilateral structures more distant from the midsagittal plane, considering the less magnified structures on the side nearby the image receptor [30]. We were not able to identify studies correlating landmark identification errors in lateral cephalograms and their influence on the outcome of patient treatment.

Finally, in 1982, De Abreu showed that different 2D cephalometric analysis may lead to different diagnosis of the same patient, varying the diagnosis between class II and class III in 8 out of 129 cases [28]. Also, Abdullah et al. [29] found that Steiner's cephalometric analysis is not accurate enough to plan orthodontic treatment. Both publications were assessed with low levels of evidence. In both publications, the number of observers was not referred. Furthermore, the statistical method used was not mentioned in [28].

The accuracy in the evaluation of the results, as well as producing changes in the treatment compared with clinical evaluation, seems to be one of the major benefits of 2D cephalometry. Risk-benefit analysis should be carefully evaluated.

## Conclusion

The existing literature suggested that lateral cephalometric radiographs have been used without adequate scientific evidence of its usefulness and are often used prior to treatment. There is a need for diagnostic accuracy studies on 2D lateral cephalometric radiograph where standardised methodological criteria for diagnostic thinking efficacy and therapeutic efficacy are incorporated. This systematic review has shown that the evidence to agree or disagree on the usefulness of this radiographic technique in orthodontics today is limited. Lateral cephalograms are used in many occasions for reasons other than clinical diagnosis or treatment, such as medico-legal reasons in a teaching environment or due to a lack of experience in the field. These conclusions are rather worrying. The use of radiation in children should be even better justified, and scientific evidence of that justification seems lacking. At present, there is a need for further studies on larger patient populations, focusing on the therapeutic efficacy of lateral cephalograms.

## Competing interests

The authors declare that they have no competing interests.

## Authors’ contributions

ARD and IBR selected and read the papers included in this systematic review. ARD drafted the manuscript. PP, SN, RO, APF and RJ participated in this systematic review by giving scientific support. All authors read and approved the final manuscript.

## Supplementary Material

Additional file 1 **Protocols 1 and 2.** The questionnaire for the initial selection of publications is shown. QUADAS-2 tool protocol was used to evaluate the methodology of included studies. Click here for file
