# Supplementary material for: Validity of 2D lateral cephalometry in orthodontics: a systematic review
Source: Prog Orthod. 2013 Sep 20;14(1):31. doi: 10.1186/2196-1042-14-31 (PMC3882109; doi:10.1186/2196-1042-14-31)
Supplement: Additional file 1 — Protocols 1 and 2. The questionnaire for the initial selection of publications is shown. QUADAS-2 tool protocol was used to evaluate the methodology of included studies. [file 2196-1042-14-31-S1.docx]

**Table S1.** Protocol 1, Selection for inclusion of publications.

| **First author:**  **Title:**  **Journal; Year; Volume; Pages:** | | | |  |
| --- | --- | --- | --- | --- |
|  | | **Yes** | **No** | |
| 1 | Is there a well-defined hypothesis? |  |  | |
| 2 | Are the accuracy, reliability, validity of cephalometry studied? |  |  | |
| 3 | Is the contribution of cephalometry in determining  the treatment plan evaluated? |  |  | |
| 4 | Reliability of landmark identification in cephalometry? |  |  | |
| 5 | Errors that occur in cephalometry? |  |  | |
| 6 | What is the level according to Fryback and Thornbury? |  |  | |
| 7 | Is the publication relevant for the review? |  |  | |

**Table S2.** Protocol 2, based on the QUADAS-2 tool for evaluation of methodology of included studies.

Observer initials ______________ Date ________

Paper nº ⁄ ______

First author; Title; Journal; Year; Volume; Pages

_______________________________________________________________________

1. Are the results of the study valid?

Yes No Unclear

2. Was the spectrum of patient’s representative of the patients who perform orthodontic treatment?

Yes No Unclear

3. Were selection criteria clearly described?

Yes No Unclear

4. Is the reference standard likely to correctly classify the target condition?

Yes No Unclear

5. Were the methods for performing the radiographic examination described in sufficient detail to permit replication?

Yes No Unclear

6. Was the execution of the reference standard described in sufficient detail to permit its replication?

Yes No Unclear

7. Were the index test results interpreted without knowledge of the results of the reference standard?

Yes No Unclear

8. Were the reference standard results interpreted without knowledge of the results of the index test?

Yes No Unclear

9. Were the same clinical data available when test results were interpreted as would be available when the test is used in practice?

Yes No Unclear

10. Were uninterpretable ⁄ intermediate test results reported?

Yes No Unclear

11. Were withdrawals from the study explained?

Yes No Unclear

12. Was the number of observers sufficient to evaluate the influence of observer reproducibility and diagnostic efficacy?

Yes No Unclear

13. Was observer reproducibility described?

Yes No Unclear

14. Were appropriate results presented (percentage of correct diagnosis, sensitivity, specificity, predictive values, measures of ROC, likelihood ratios, or other relevant measures) and were these calculated appropriately?

Yes No Unclear

Comments
